# A novel de Novo KCNC1 mutation (c.1147 C > T) presenting with epilepsy and ADHD: a case report and literature review

**DOI:** 10.1186/s12883-026-04677-z

**Published:** 2026-02-02

**Authors:** Chuyu Huang, Yongyan Kang, Runxin Peng, Haoyuan Zhong, Ningjing Zeng, Linzhu Zhang, Xinying Chen, Shujuan Du

**Affiliations:** 1 The Fifth Clinical College of Guangzhou University of Chinese Medicine, Guangzhou, China; 2 Guangdong Provincial Second Hospital of Traditional Chinese Medicine, Guangzhou, China; 3 The Second Clinical College of Guangzhou University of Chinese Medicine, Guangzhou, China; 4 The Second Affiliated Hospital of Guangzhou University of Chinese Medicine, Guangzhou, China; 5 Shenzhen Children’s Hospital, Shenzhen, China; 6 Lingnan Pediatrics Wenziyuan Academic School Inheritance Studio, Guangdong Provincial Hospital of Chinese Medicine, Guangzhou, China

**Keywords:** KCNC1, Epilepsy, Neurological disorder, Gene mutation, Case report

## Abstract

**Background:**

Pathogenic KCNC1 mutations (encoding Kv3.1 potassium channels) drive heterogeneous neurological disorders, ranging from progressive myoclonus epilepsy-ataxia (MEAK) to developmental/epileptic encephalopathies (DEE) and global developmental delay. Transmembrane-domain variants predominantly cause MEAK-like phenotypes, whereas cytoplasmic mutations associate with severe DEE characterized by refractory seizures and cognitive impairment. The genotype-phenotype correlation in the currently reported 54 cases remains unclear, particularly for non-transmembrane mutations. This paper describes a novel KCNC1 variant (c.1147 C > T, p.His383Tyr) and utilizes protein modeling to elucidate its structural impact, aiming to advance precision therapy for KCNC1-related channelopathies.

**Case presentation:**

An 11-year-old male with normal early development developed febrile convulsions at 19 months, progressing to generalized tonic-clonic seizures and drop attacks by 20 months. Initiated on valproate at age 2 after EEG confirmation of epilepsy, he achieved sustained seizure freedom (> 3 years) with dose optimization. Current assessment shows age-appropriate motor/social function and superior cognition (Raven’s 75-80th percentile) alongside idiopathic short stature (-2.08 SDS height), persistent EEG abnormalities, mega cisterna magna on MRI, and Attention-Deficit/Hyperactivity Disorder-related academic impairment. The whole exome sequencing identified a de novo KCNC1 mutation (c.1147 C > T). Three-dimensional protein modeling demonstrated structural disruption in voltage-sensing domains. Comparative analysis of 54 published cases revealed that transmembrane domain mutations predominantly cause epilepsy-ataxia syndrome, whereas non-transmembrane variants are correlated with developmental encephalopathies.

**Conclusions:**

The present study is the first to report a c.1147 C > T KCNC1 mutation and highlights the importance of transmembrane domain integrity for neurological function. The dissociation between seizure control and persistent neurodevelopmental deficits suggests distinct pathomechanisms for epileptic versus cognitive manifestations. These findings emphasize the prognostic value of mutation localization as well as support early genetic testing in neurological disorder patients with subclinical EEG abnormalities.

**Supplementary Information:**

The online version contains supplementary material available at 10.1186/s12883-026-04677-z.

## Background

Pathogenic heterozygous mutations in the potassium voltage-gated channel subfamily C member 1 (KCNC1, encoding Kv3.1) are associated with diverse neurological disorders [1]. These mutations were initially described by Muona et al. (2015) in patients with progressive myoclonus epilepsy (PME) and ataxia, referred to as “Myoclonus Epilepsy and Ataxia due to Potassium Channel Mutation” (MEAK). At present, they have been demonstrated to be responsible for broader clinical spectrum [1–3]. KCNC1 variants may lead to severe neurological conditions including developmental and epileptic encephalopathy (DEE), characterized by early-onset refractory seizures, developmental delay, and cognitive impairment, particularly when mutations occur in cytoplasmic domains [1, 4]. In 2019, Cameron first described KCNC1-related DEE and demonstrated through experimental studies that loss-of-function is a key feature [5]. Additionally, KCNC1-related conditions such as global developmental delay (GDD), intellectual disability (ID), developmental encephalopathy (DE), and epilepsy have also been reported [1, 5–7].

The KCNC1 gene encodes a Kv3.1 voltage-gated channel found primarily in fast-spiking neurons of the central nervous system, especially in inhibitory GABA interneurons. This channel achieves high-frequency firing through fast activation and membrane repolarization [2]. KCNC1 mutations can damage and impair the physiological function of GABAergic inhibitory interneurons and cerebellar neurons. Lesions in the former cause myoclonus and generalized tonic-clonic seizures (GTCS), whereas those in the latter can lead to ataxia and tremor [4]. Notably, transmembrane-domain mutations typically cause disorders with PME, while non-transmembrane mutations correlate with non PME phenotypes [1, 3]. Approximately 50 cases of KCNC1-related neurological disorders have been documented [8]. In this paper, we report a case of a unique genotype and compare it with previous cases. Consequently, we aimed to analyze the harm caused by the KCNC1 mutation through the construction of protein modeling. This approach aims to elucidate genotype-phenotype correlations across the KCNC1 mutation spectrum. Our findings provide a framework for developing precision treatments for KCNC1-related neurological disorders.

## Case presentation

As the third of four siblings, the proband is a male child born at term via natural delivery in February 2012 without neonatal asphyxia or jaundice. His developmental milestones are within normal ranges: he started walking independently at 13 months and demonstrates age-appropriate language skills. Family history noted no epilepsy in first-degree relatives, though a maternal aunt had febrile convulsions. He experienced a febrile convulsion at 19 months and seizures characterized by GTCS at 20 months. Beginning before age 2 years, he developed recurrent paroxysmal bruxism and unprovoked drop attacks, necessitating evaluation at a pediatric neurology center. A short-range EEG revealed abnormal epileptiform discharges, confirming the diagnosis of epileptic seizures. valproic acid (0.2 g QD) therapy was thus commenced at age 2. Subsequent doses were adjusted incrementally based on weight gain and therapeutic drug monitoring, which resulted in complete resolution of seizures.

**Fig. 1:**
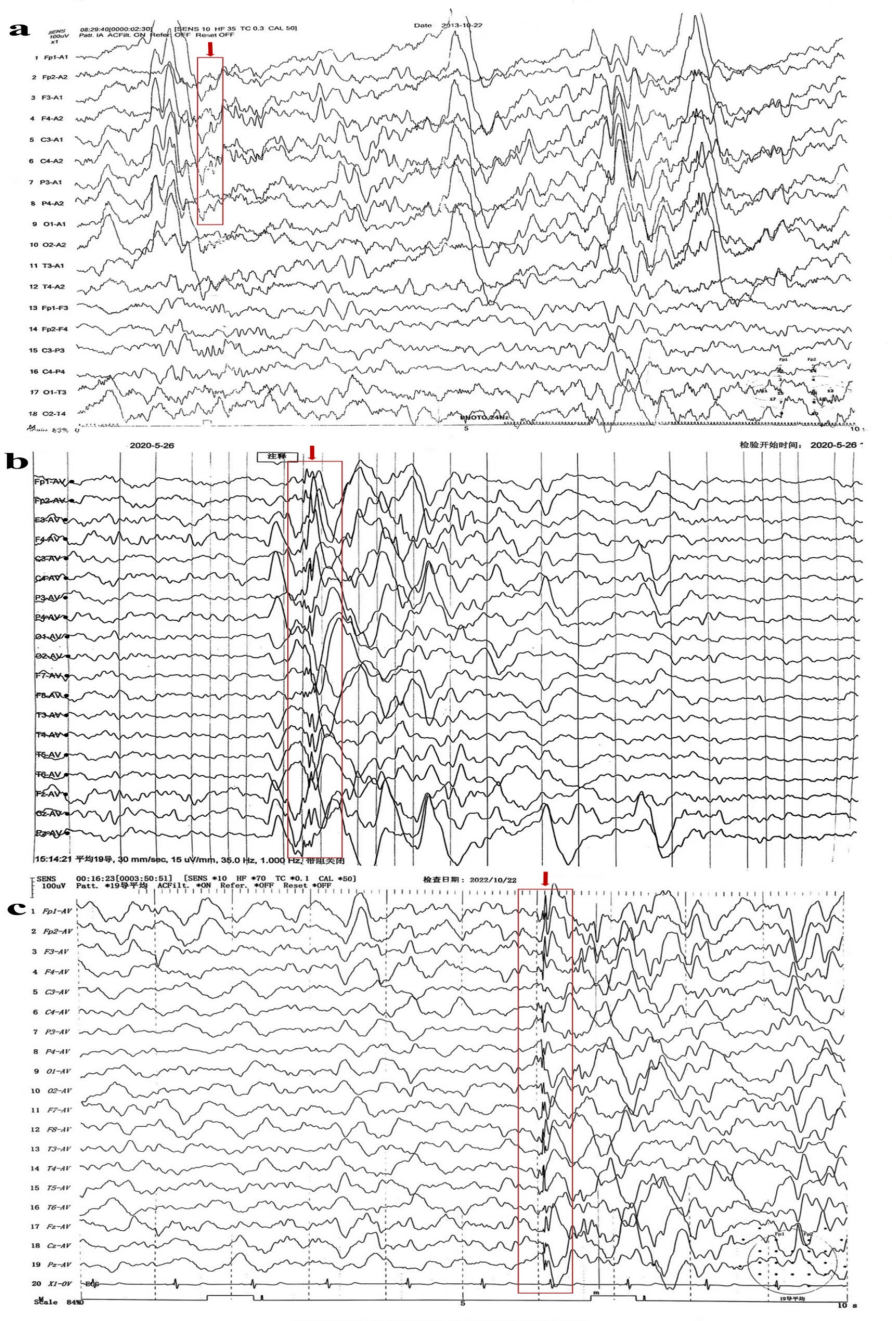
**a** 2013-10-22 EEG; the time indicated by the arrow shows shape waves. **b** 2020-05-26 EEG; the time indicated by the arrow shows shape waves. **c** 2022-10-22 EEG; the time indicated by the arrow shows multispike-slow waves

**Fig. 2:**
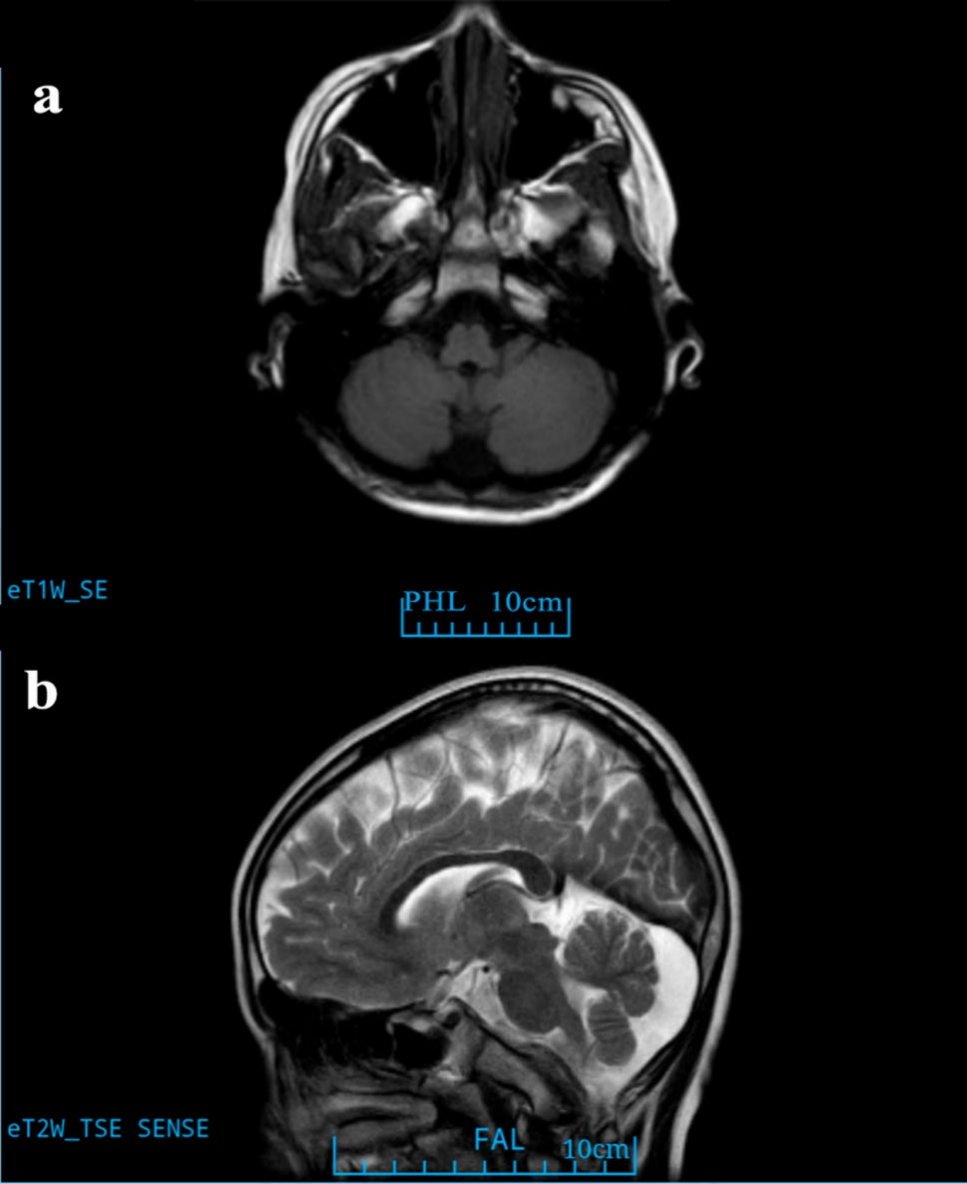
1.5T MRI (**a**) axial T1 and (**b**) sagittal T2 image showing mega cisterna magna at the age of 10

Upon assessment in July 2025 (at the age of 13 years), he exhibited normal gait and age-appropriate motor function, including the ability to jump vertically. Social interactions with peers were developmentally appropriate. Currently attending sixth grade, his academic performance is below average due to Attention-Deficit/Hyperactivity Disorder, predominantly inattentive presentation (ADHD-PI) diagnosed in 2022. A physical examination conducted in June 2025 documented a height of 147 cm (2nd percentile, −2.08 SDS) and a weight of 35.5 kg (6th percentile, −1.57 SDS), which is consistent with idiopathic short stature (ISS). Neurological assessment indicated normal muscle strength/tone, intact physiological reflexes, absent pathological reflexes, and negative tests for ataxia despite mild gait unsteadiness. Diagnostic testing included: Annual EEG monitoring revealed persistent epileptiform abnormalities (Table 1; Fig. 1a-c). 1.5T brain MRI demonstrating mega cisterna magna (Fig. 2) with normal cerebellar volume and cortical architecture (Fig. S2); normal bilateral upper limb EMG; and a Raven’s Progressive Matrices score of 48 (indicating above-average intellectual ability, placing him between the 75th and 80th percentiles). Throughout the three-year follow-up period, he experienced no progression of symptoms.

**Table 1:** Electroencephalogram (EEG) monitoring results of the patient

Time (Year-month)	EEG duration (hours)	EEG results
2013-10	2	Multiple episodes of high amplitude slow waves and slow activity were observed in each lead. The paroxysmal slow waves were increased under flash stimulation, and some of them changed into sharp waves.
2015-02	2	Each lead can be seen as a single or several episodes of 2.0–4.7 Hz slow wave and slow activity. Increased paroxysmal slow wave, slow activity and sharp wave under flash stimulation.
2020-05	2	Abnormal epileptiform discharge during sleep: spikes, sharp waves and sharp slow waves emanate from the left frontal area; The bilateral prefrontal area has slightly more small spike clusters.
2022-04	2	Abnormal epileptiform discharge during sleep: a multispike wave mainly on the left frontocentral area, spreading to the adjacent leads of the same side and the opposite side.
2022-10	24	Normal background, few paroxysms of widespread irregular spikes and slow waves, multispike-slow wave, slow activity at 4–5 Hz during sleep.
2024-07	24	Normal background. A single generalized multispike-slow wave array was observed during sleep, and occasionally (twice) sharp wave emission was observed in the left temporal area (the abnormal wave was reduced compared with the previous figure).
2025-07	24	Normal background. Interictal sleep periods showed occasional epileptiform discharges in the left anterior head regions and right frontotemporal regions, along with generalized spikes and slow waves, multispike-slow wave.

**Fig. 3:**
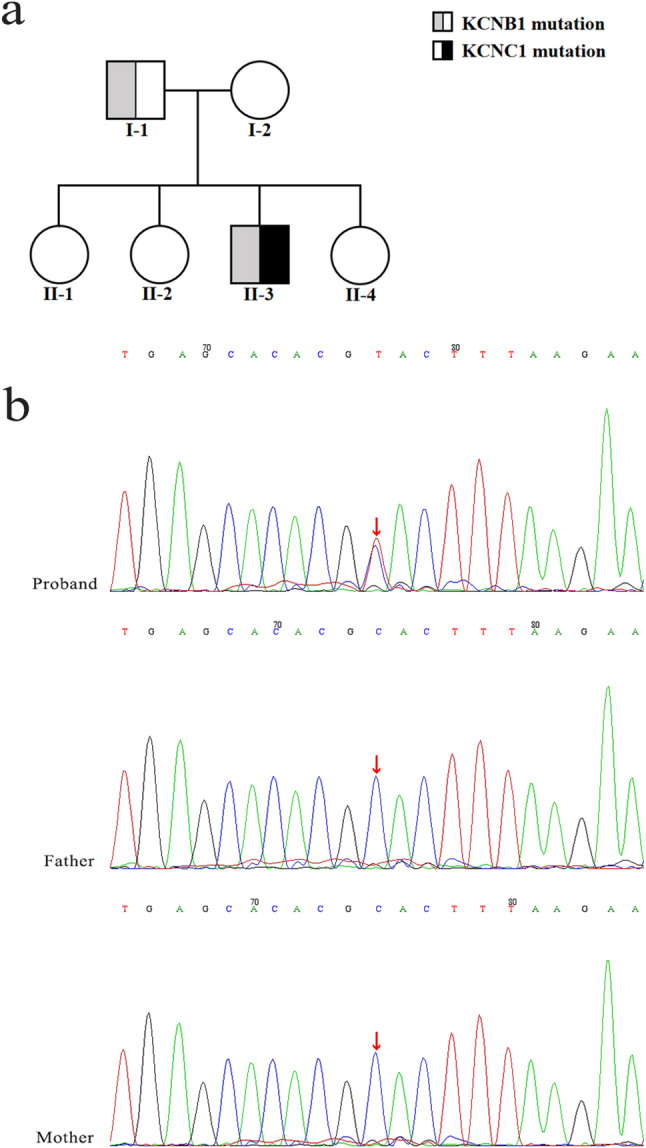
**a** Pedigrees of the family. The II-3 is the proband. **b** The Sanger sequencing results of patient families with KCNC1 mutations

For whole genome sequencing, high-throughput sequencing analysis was performed. Peripheral venous blood (2 mL) was collected from both the proband and his parents and stored in an ethylenediaminetetraacetic acid anticoagulant tube. The samples were sent to the medical laboratory (MyGenomics, Beijing) to complete subsequent operations. All the variants were confirmed by Sanger sequencing. The results of exome sequencing were as follows: (1) There was a de novo heterozygous mutation in the KCNC1 gene, chromosomal location chr11:17793788, exon NM_001112741.2; exon 2, nucleotide position c.1147 C > T, amino acid position p.His383Tyr. Family verification analysis revealed that there was no variation in this site between the parents (Fig. 3a-b). The corresponding disease is progressive myoclonic epilepsy-7 (OMIM: 616187), and the inheritance mode is autosomal dominant. According to the American College of Medical Genetics and Genomics Guidelines (ACMG), the variant is preliminarily identified as Likely pathogenic [9]: ①PM2_Supporting: The frequency in the normal population database (gnomAD_exome ALL, gnomAD exome East Asian) is “-”; ② PP2: This gene rarely has benign missense variation, which is a common mechanism of disease; ③ PS2: According to family verification analysis, there was no variation in the site of his parents, which was a de novo mutation. The correlation of this site was not reported in the literature database. Also, pathogenicity analysis of this site was not found in the ClinVar database. The prediction result of REVEL was uncertain, whereas the prediction results of other online prediction sites were mostly benign, as shown in Table 2 [9–11]. (2) Heterozygous mutation in the KCNB1 gene, chromosomal location chr20:47989647, exon NM_004975.4; exon 2, nucleotide position c.2450 C > G, amino acid position p.Thr817Arg. According to the family verification analysis, the source of variation was the father (heterozygote) and the mother of the child had no variation at this site (Fig. 3a, Fig. S1). The corresponding disease is developmental and epileptic encephalopathy-26 (OMIM: 616056), whose inheritance mode is autosomal dominant. According to ACMG, KCNB1 variation is preliminarily determined to be Uncertain [9]: ① BP4: REVEL predicts that the results are probably benign, whereas the other websites predict that the results are mostly benign, as shown in Table 2 [9–11]. ② PP2: This gene rarely has benign missense variation, which is a common mechanism of disease. The correlation of this site was not reported in the literature database, and pathogenicity analysis of this site was not found in the ClinVar database. The frequency in the normal population database is 0.0006427. According to family verification analysis, the father of the subject had heterozygous variation at the site, whereas the mother of the subject had no variation.

**Table 2:** Pathogenicity prediction using in Silico tools

No.	In silico analysis tools	KCNC1 (*p*.His383Tyr)		KCNB1 (*p*.Thr817Arg)	
		Prediction	Score	Prediction	Score
1	SIFT	Tolerable	0.384	Tolerable	0.276
2	Polyphen-2	Benign	0.004	Benign	0.014
3	CADD	Tolerable	6.898	Benign	< 0.15
4	REVEL	U	0.463	LB	0.259
5	Mutation Taster	Disease_causing	1.000	Polymorphism	0.957

**Fig. 4:**
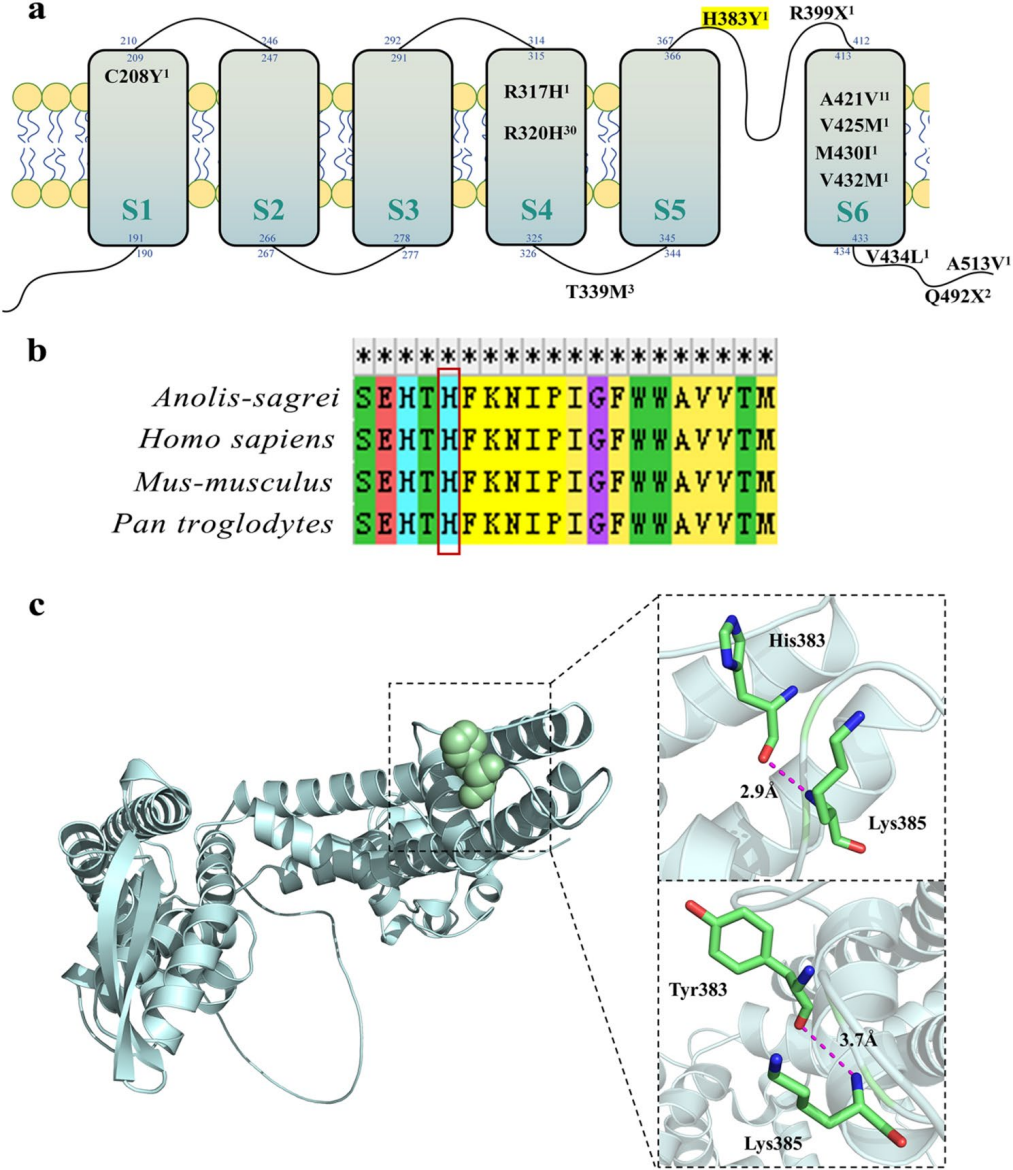
**a** Schematic diagram of the inferred variation location of the Kv3.1 channel reported in this study. The double rows of yellow spheres represent the membrane structure, and the six cuboids represent the six transmembrane helices (S1–S6). The blue number is the sequence of amino acids separated within, across and outside the membrane. The black letter indicates the relative sequence position of the existing mutant amino acid site and the amino acid abbreviation before and after mutation. The right superscript indicates the number of reported cases. H383Y, namely p.His383Tyr, is the mutation reported in this study. **b** Multiple sequence alignments of residues, with the red box highlighting the location of the missense variant at His383. Sequence two is homo sapiens. **c** Complete 3D model structure of the KCNC1 protein and the predicted 3D structure of wild-type KCNC1 and mutant KCNC1

The following species amino acid sequences were downloaded from the NCBI website (https://www.ncbi.nlm.nih.gov): *Homo sapiens*, *Pan troglodytes*, *Mus-musculus*, *Anolis-sagrei*. MEGA 11.0 software was used to compare the amino acid sequences of KCNC1 in different species [12]. The amino acid sequences of KCNC1 in different species were compared. The analysis involved four amino acid sequences. All fuzzy positions of each sequence pair are removed (pair deletion option). As shown in Fig. 4b, this residue site is highly conserved.

The three-dimensional structure of the KCNC1 protein, along with both the wild-type and mutant configurations, was computationally modeled via the SWISS-MODEL online platform and visualized via PyMOL software (version 3.1), as depicted in Fig. 4c [13]. The wild-type protein is a histidine with a molecular weight of 155 kDa, and the side chain containing an imidazolyl group, which is positively charged under neutral conditions, is an alkaline amino acid. When mutated to tyrosine, the molecular weight is 181 kDa. Its side chain is an aromatic ring, and it is a neutral amino acid without charge. After mutation, the hydrogen bond distance to the 384th amino acid increases.

## Discussion and conclusions

### Literature review and clinical characteristics

The literature search was conducted on the Chinese National Knowledge Infrastructure, Wanfang Database and PubMed from the inception date to June 2024. The search strategies employed Boolean operators to combine core terms: epilepsy, KCNC1, progressive myoclonic epilepsy (PME), developmental and epileptic encephalopathy (DEE) and neurological disorder, using synonymous expressions for each term. Two investigators independently screened titles/abstracts using predefined criteria: (1) human studies; (2) reported KCNC1 variants with phenotypic data; (3) full-text availability. Discrepancies were resolved by consensus. A total of five Chinese studies reporting seven KCNC1 cases were included [8, 14–17]. Ten English studies reporting 47 KCNC1 cases were included [1, 3, 5–7, 18–22]. A total of 55 cases were reported in this paper. The literature review flow chart is shown in Fig. S2. The clinical characteristics and genetic test results of all patients are shown in Table S1.

This study analyzed 55 patients (28 male, 25 female; 2 unspecified) aged 1–63 years. Disease onset ranged from birth to 15 years, with initial symptoms dominated by myoclonic seizures (MS, 40.0%, 22/55), GTCS (36.4%, 20/55), tremor (20.0%, 11/55) and ataxia (3.6%, 2/55). During disease progression, 81.8% (45/55) developed epilepsy, primarily manifesting as MS (84.4%, 38/45) and GTCS (80.0%, 36/45); 77.8% (35/45) exhibited ≥ 2 seizure types, while 9 patients (16.4%) presented developmental delay without epilepsy.

Neurological examination revealed ataxia in 72.7% (40/55) and hypotonia in 29.1% (16/55), with additional signs including mild weakness, nystagmus, and cortical visual impairment. Developmental abnormalities included developmental delay (45.5%, 25/55), motor regression (1.8%, 1/55), intellectual disability (30.9%, 17/55), learning difficulties (10.9%, 6/55), Attention-Deficit/Hyperactivity Disorder (ADHD) (5.5%, 3/55) and cognitive decline (30.9%, 17/55).

Notable ancillary features comprised febrile seizure history (9.1%, 5/55) and symptom improvement during fever (12.7%, 7/55). Craniofacial dysmorphisms (14.5%, 8/55) included four phenotypic profiles: (1) happy demeanor, dysconjugate gaze, bilateral downslanting palpebral fissures, high-arched palate (*n* = 1); (2) Macrostomia, smooth philtrum, upslanting palpebral fissures, dental enlargement (*n* = 3); (3) Epicanthal folds, ptosis, short philtrum, prognathism, persistent fetal finger pads, prominent ears (*n* = 3); (4) Hypertelorism, long palpebral fissures, broad nose, large ears, diastema, micrognathia (*n* = 1). Musculoskeletal/systemic features (5.5%, 3/55) included three phenotypic profiles: (1) Dorsal hirsutism, slender extremities, joint hyperextensibility, pes planus (*n* = 1); (2) Joint hypermobility (*n* = 1); (3) Congenital right talipes equinovarus (*n* = 1).

Genetic evaluation identified 13 familial cases (23.6%) from five KCNC1 families and 42 sporadic cases (76.4%). Among the sporadic cases, one had a family history of epilepsy and another had a family history of febrile seizures. Co-occurring non-KCNC1 mutations were detected in 12.7% of patients (7/55). Diagnostic investigations showed epileptiform discharges in 91.8% (45/49) of EEGs, while brain MRI abnormalities (39.1%, 18/46) were predominantly cerebellar atrophy (83.3%, 15/18), including one progressive case.

Regarding treatment, antiseizure regimens included ≤ 2 drugs (40.0%, 22/55), ≥ 3 drugs (30.9%, 17/55), and adjunctive tremor control (14.5%, 8/55). In 20 documented cases, GTCS control rates were 35.0% (achieved control), 45.0% (improved), and 20.0% (refractory); MS control rates were 20.0%, 60.0%, and 15.0%; corresponding EEG control rates were 33.3% (achieved control), 33.3% (improved), and 33.3% (refractory). Four patients (20.0%) were medication-refractory, while one responded to vagus nerve stimulation. Functional outcomes included independent living (20.0%, 11/55), semi-dependence (10.9%, 6/55), and full dependence (38.2%, 21/55). Two patient fatalities occurred (3.6%): one 63-year-old patient died due to respiratory failure and one 6-month-old patient died due to cardiac arrest.

### Correlation between clinical manifestations and transmembrane localization of mutation sites in patients

**Fig. 5:**
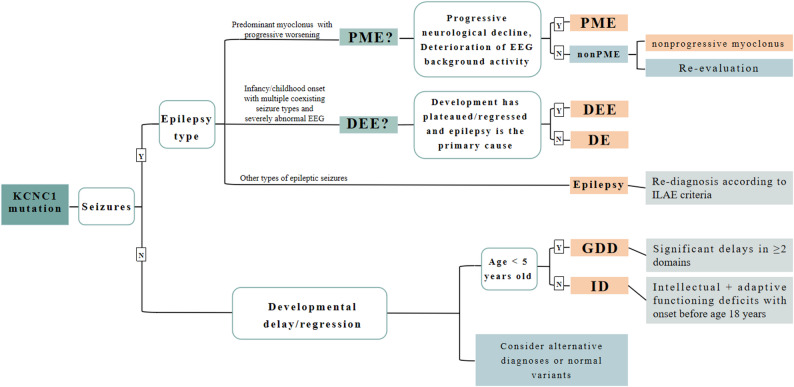
Diagnostic algorithm for KCNC1-mutated cases synthesized from literature evidence. *PME* Progressive myoclonus epilepsy, *GDD* Global developmental delay, *ID* Intellectual disability, *DEE* Developmental and epileptic encephalopathy, *DE* Developmental encephalopathy

The TMHMM website was used to predict the amino acid transmembrane region, and the schematic diagram in Fig. 4a was drawn on the basis of previous literature reports [23]. The blue numbers indicate the sequence of the amino acid intramembrane, transmembrane region and extramembrane separation. In this study, we report a new case of the mutation c.1147 C > T (p.His383Tyr) and we observed that p.His383Tyr was located in the junction region of S5 and S6. Previous studies and the current findings identified 13 mutation sites in total. Seven mutation sites (46 cases) were located in the transmembrane domains, including one in the S1 region, two in the S4 region, and four in the S6 region. Six mutation sites (nine cases) were located in the non-transmembrane domains, including four in the intramembrane regions and two in the extramembrane regions. The most frequently reported mutation site was p.Arg320His (30 cases). The relationship between KCNC1 gene mutation and clinical manifestations may depend on whether the mutation site is located in the transmembrane domains. The distribution of confirmed KCNC1 mutations in transmembrane and non-transmembrane domains among associated disorders is presented in Table 3; Fig. 5.

**Table 3:** Distribution of confirmed cases with KCNC1 mutations in transmembrane versus non-transmembrane domains across associated disorders​

Mutation sites		Transmembrane domain mutation *n* (%)	Non-transmembrane mutation *n* (%)
Cases		46 (83.6)	9 (16.4)
Diagnosis	PME	31 (56.4)	0
	DEE	10 (18.2)	1 (1.8)
	GDD	2 (3.6)	1 (1.8)
	DE	1 (1.8)	2 (3.6)
	ID	0	4 (7.3)
	Epilepsy	1 (1.8)	1 (1.8)
	nonprogressive myoclonus	1 (1.8)	0

*PME* Progressive myoclonus epilepsy, *GDD* Global developmental delay, *ID* Intellectual disability, *DEE* Developmental and epileptic encephalopathy, *DE* Developmental encephalopathy

The data in Table S1 and Fig. 4a suggest that the main manifestation of the mutation in the transmembrane domains was typical epilepsy (93.5%, 43/46). Among them, 87.0% (40/46) of patients reported ataxia, 26.1% (12/46) of patients reported hypotonia, and 41.3% (19/46) of patients reported developmental retardation. Approximately 87% of patients had abnormal EEGs with epileptiform discharges. Cerebellar atrophy was indicated by brain MRI in 32.6% (15/46) of the patients. 43.6% of patients (17/39) were treated with three or more drugs and were classified as drug-resistant epilepsy [24]. In terms of prognosis, 83.3% of patients (15/18) had controlled/improved epilepsy symptoms after treatment; however, drugs could not prevent the progression of ataxia. Other manifestations, such as ID and dystonia were still observed. In addition, patients with transmembrane gene mutations primarily presented with non-seizure-related symptoms as their initial clinical manifestation: Clatot reported two cases of global motor delay and central hypotonia as the first presentation [1], and Cameron reported a case of developmental encephalopathy as the first presentation [5]. For patients with mutations in non-transmembrane domains, the main manifestations were developmental encephalopathy or developmental delay, with one patient presenting nystagmus. No consistent clinical pattern was observed among these cases. None of the patients reported ataxia. Three patients had abnormal EEGs, two patients had normal-abnormal EEGs under dynamic monitoring, and two patients’ EEGs were normal. Their brain MRI scans were normal.

To date, our patient has been diagnosed with neurological disorder, ADHD-PI and ISS without clinical symptoms of cerebellar lesions. The mutation site (p.His383Tyr) was located in the non-transmembrane domain between S5 and S6. Compared with those of other reported patients with non-transmembrane domains, several of the following clinical characteristics are similar: early onset time, abnormal EEG findings and normal brain MRI findings. However, our patient developed GTCS and myoclonus at approximately two years of age, and the symptoms were completely controlled after taking valproic acid. The above characteristics are inconsistent with the previously reported pathogenesis of non-transmembrane mutations. A special patient who presented with Epilepsy of Infancy with Focal Migrating Seizures (EIFMS) died at six months of age. No significant association was observed between KCNC1 and the onset of EIFMS [5]. Regarding ADHD, a population-based cohort study has shown that the prevalence of ADHD in epilepsy is higher than that in normal children, suggesting that common neurobiological mechanisms may be present in epilepsy and ADHD [25]. However, with only three cases of ADHD reported in this study, a clear association between ADHD and KCNC1 mutations could not be established, and this potential link requires further investigation [19, 21]. No cases of epilepsy have been reported in children with early manifestations, such as developmental delay. They may not have had epilepsy, or they may not have been observed long enough to progress. Therefore, we can infer that variations in regions with conductive conformational functions cause severe epilepsy phenotypes, whereas variations in other areas may be associated with mild symptoms.

### Mechanisms of KCNC1 mutations and exploration of potential therapeutic approaches

Potassium (K^+^) voltage-gated channels (K_V_), the largest family of potassium (K^+^) channels, are distributed mainly in the central nervous system and are involved in the regulation of the cell membrane resting potential and action potential repolarization to determine the frequency and amplitude of action potentials [26]. Kv3.1 is a tetramer of four α-subunits, each containing six transmembrane helices (S1-S6). Among them, the S4 segment forms the main voltage sensor, whereas the S5 and S6 segments form the central ion pore [27, 28]. Kv3 channels usually open only at positive potentials and respond rapidly [26]. Some previous reports have indicated that transmembrane protein mutations in the KCNC1 gene mainly affect the electric current. In contrast, non-transmembrane channel protein mutation exhibits no clear pattern; for example, transmembrane variations such as p.Arg320His and p.Ala421Val can lead to a significant reduction in the electric current amplitude [5]. Zhang concluded that the electric current of A513V was almost the same as that of wild type, but the mutation prevented the phosphorylation of the channels by protein kinase C [29]. Our study indicates that the KCNC1 mutation in this study may also affect the repolarization and hyperpolarization of the action potential via the above mechanism, so that the cell’s excitability cannot be reduced normally, resulting in the occurrence of MS. The mutation sites reported in this paper have strongly conserved amino acids. Mutation of histidine to tyrosine at this site increases the molecular weight and introduces steric hindrance to neighboring amino acids. The side chain changes from heterocyclic to aromatic, increasing hydrophobicity and decreasing the isoelectric point. These modifications alter the protein folding and increase the hydrogen bond distances [30]. Further studies are warranted to investigate the effect of spatial structure changes on the function of KCNC1 mutants.

The functional changes caused by Kv3.1 gene mutation may be related to changes in the electric current and voltage. In the study of targeted therapy, the positive regulation of Kv3.1 and Kv3.2 electric currents by the small-molecule activators EX15 and RE01 of Kv3 channels promoted an increase in the firing frequency of fast peak GABAergic inhibitory interneurons [31]. EX15 and RE01 can improve the progressive dysfunction of neuronal network dynamics by activating targeting fast-spiking interneuron activity [32]. RE01 partially improves electric current reduction and slow activation kinetics. These effects suggest that activated Kv3.1 may have the potential to reduce epilepsy severity and improve cognitive function [33]. With the wide application of genetic testing, it has played an increasingly important role in the field of epilepsy and can better assist in clinical diagnosis and treatment and in evaluating patient prognosis. Early genetic testing could contribute to determine appropriate treatment plans and improve patient prognosis [34].

In summary, we identified a novel heterozygous KCNC1 variant. Its phenotypic characteristics are unique compared with those of some previously reported patients, and this variant needs to be followed up. Additionally, this study summarized existing KCNC1-related neurological disorders and inferred the similarities and differences in clinical manifestations on the basis of whether mutation sites were located in the transmembrane domain. No electrophysiological validation has been performed. Besides, further investigation is required to understand the mechanism by which KCNC1 mutations at this site lead to clinical presentations and the differences among mutation sites.

## Supplementary Information

Supplementary Material 1.

Supplementary Material 2.

## Data Availability

All the literature reviewed in this study is included in Supplementary Table 1. The datasets generated and/or analysed during the current study are available in the Genome Sequence Archive repository, HRA010421 (https://ngdc.cncb.ac.cn/gsa-human/). The other patient data used in this study are available from the corresponding author on reasonable request.

## Abbreviations

ACMG: the American college of medical genetics and genomics guidelines
ADHD: Attention-Deficit / Hyperactivity Disorder
ADHD-PI: Attention-Deficit / Hyperactivity Disorder, predominantly inattentive presentation
DE: Developmental encephalopathy
DEE: Developmental and epileptic encephalopathy
EIFMS: Epilepsy of infancy with focal migrating seizures
EEG: Electroencephalogram
GDD: Global developmental delay
GTCS: Generalized tonic-clonic seizures
ISS: Idiopathic short stature
KCNC1: Potassium voltage-gated channel subfamily C member 1
K_V_: Potassium voltage-gated channels
K_V_3.1: Potassium voltage-gated channel subfamily C member 1
MEAK: Myoclonus epilepsy and ataxia due to potassium channel mutation
MRI: Magnetic resonance imaging
MS: Myoclonus
PME: Progressive myoclonus epilepsy
VNS: Vagus nerve stimulation
