# Supplementary material for: A novel de Novo KCNC1 mutation (c.1147 C > T) presenting with epilepsy and ADHD: a case report and literature review
Source: BMC Neurol. 2026 Feb 2;26:141. doi: 10.1186/s12883-026-04677-z (PMC12954995; doi:10.1186/s12883-026-04677-z)
Supplement: Supplementary file 2 — Supplementary Material 2. [file 12883_2026_4677_MOESM2_ESM.docx]

**
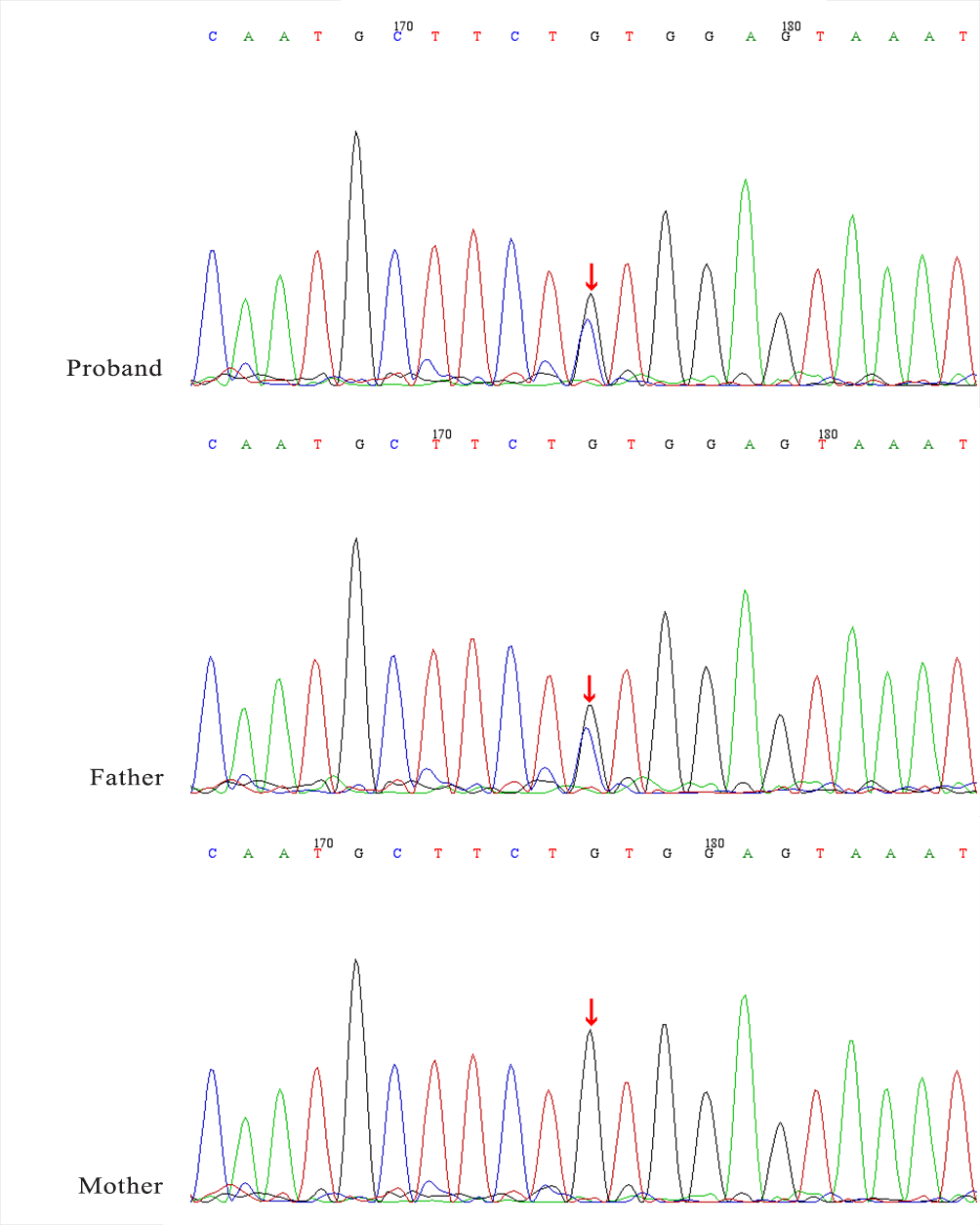
**

**Fig. S1** Sanger sequencing results of patient's families with KCNB1 mutations


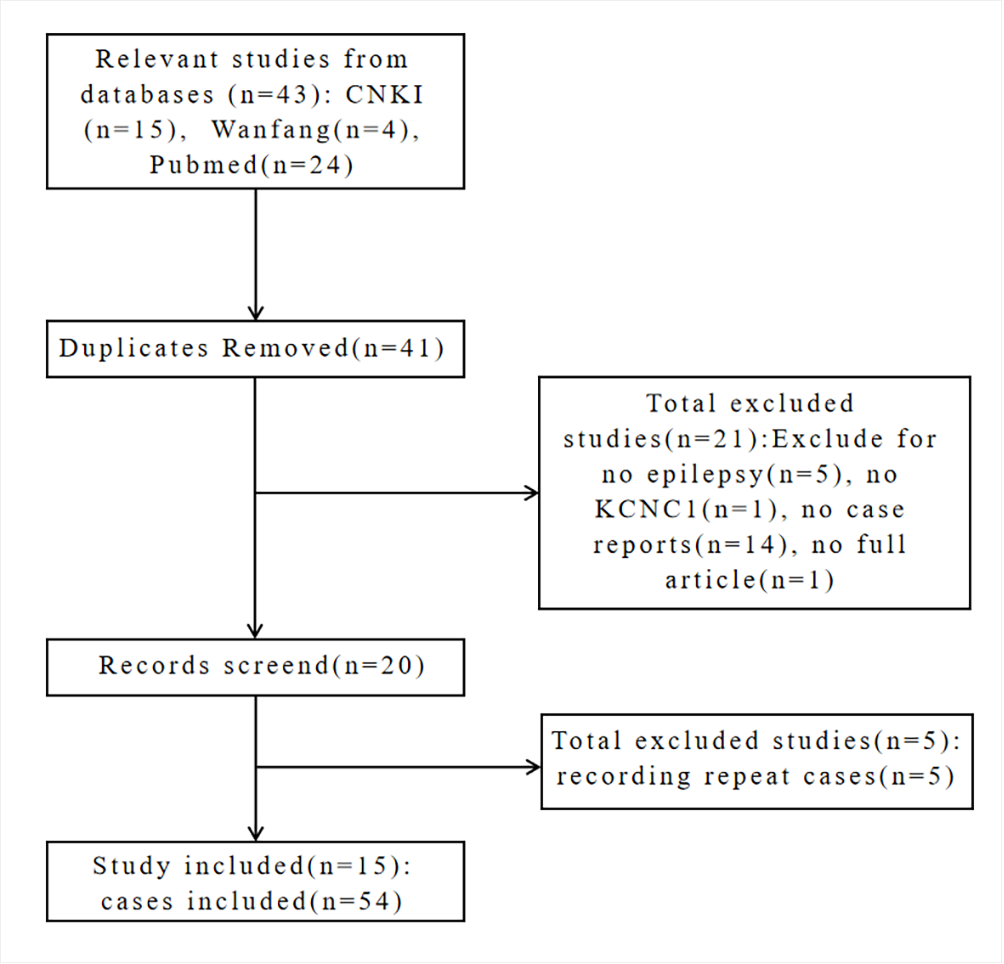


**Fig. S2** Flow diagram of detailed search strategies
